# Research progress of circRNAs in bone-related diseases

**DOI:** 10.3389/fonc.2025.1481322

**Published:** 2025-01-27

**Authors:** Xianming Hua, Lingfeng Yu, Hao Zhu, Yan Zhu, Gentao Fan, Guangxin Zhou

**Affiliations:** ^1^ Department of Orthopedics, Jinling Hospital, Affiliated Hospital of Medical School, Nanjing University, Nanjing, China; ^2^ Department of Orthopedic Oncology, Shanghai Bone Tumor Institute, Shanghai General Hospital, Shanghai Jiao Tong University School of Medicine, Shanghai, China; ^3^ School of Life Sciences, Nanjing University, Nanjing, Jiangsu, China; ^4^ State Key Laboratory of Pharmaceutical Biotechnology, Nanjing University, Nanjing, Jiangsu, China; ^5^ Wuxi Xishan Nanjing University (NJU) Institute of Applied Biotechnology, Wuxi, Jiangsu, China

**Keywords:** circRNAs, non-coding RNAs, orthopedic diseases, therapeutic target, biomarker

## Abstract

Circular RNAs (circRNAs) are non-coding RNAs that exist naturally in various eukaryotic organisms. The majority of circRNAs are produced through the splicing of exons, although there are a limited number that are generated through the circularization of introns. Studies have shown that circRNAs play an irreplaceable role in the pathogenesis, disease progression, diagnosis, and targeted therapy of motor system tumors (osteosarcoma), metabolic diseases (osteoporosis), and degenerative diseases (osteonecrosis of the femoral head, osteoarthritis, intervertebral disc degeneration). This review summarizes the advancements in circRNA detection techniques and the research progress of circRNAs in orthopedic diseases.

## 1 Introduction

**Figure 1:**
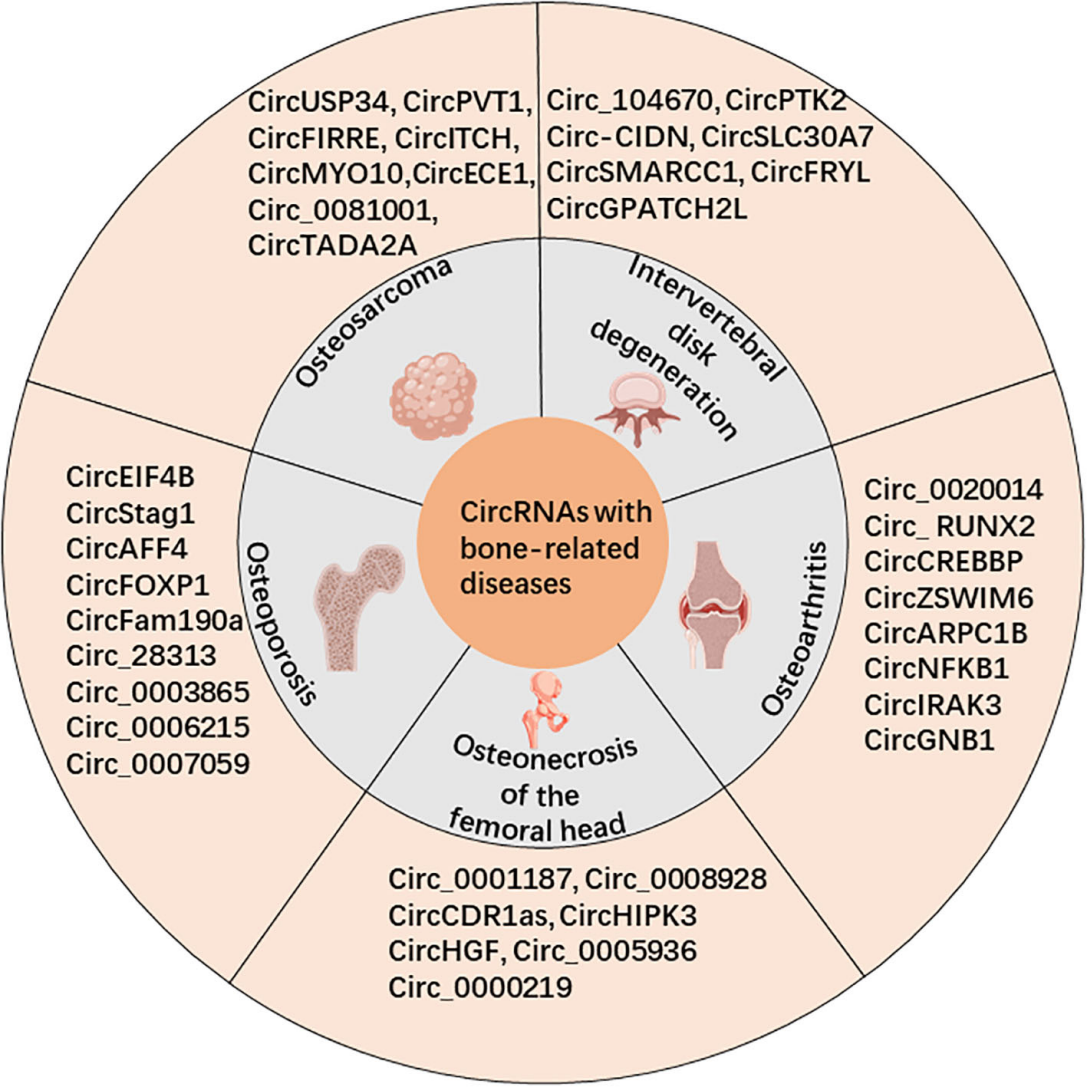
CircRNAs are associated with bone-related diseases, including osteosarcoma (OS), osteoporosis (OP), osteonecrosis of the femoral head (OFNH), osteoarthritis (OA), and intervertebral disc degeneration (IDD).

Circular RNAs (circRNAs) are a unique subclass of noncoding RNA characterized by their covalently closed loop structure, which connects from the 3’ to 5’ end. The existence of circRNAs was first confirmed by electron microscopy in 1976 in viroids and Sendai viruses by Sanger and Kolakofsky et al. (1, 2). However, due to limitations in detection techniques and research methods, circRNAs were initially believed to be low-abundance nonfunctional byproducts or “splicing noise” (3). With the advent of high-throughput sequencing technology (RNA-seq) and bioinformatics, it has been demonstrated that circRNAs are highly conserved and widely expressed in numerous cells and tissues (4–6). In fact, Guo (7) and colleagues discovered that over 10% of the tested cells and tissues’ expressed genes could generate circRNAs. Recent research has revealed that circRNAs may function through the following ways: (i) regulating gene transcription and RNA splicing (8, 9); (ii) regulating gene expression post-transcriptionally by competitively binding miRNAs through the competitive endogenous RNA (ceRNA) mechanism (10, 11); (iii) binding to RNA-binding proteins (RBPs) to modulate their activity (12); and (iv) encoding functional peptides directly (13). Recent studies have established the atypical expression of circRNAs in different diseases, including neoplasms like lung cancer (14), liver cancer (15), gastric cancer (16), and osteosarcoma (OS) (17). Additionally, circRNAs have been found to be involved in cardiovascular diseases (18), respiratory diseases (19), and reproductive system diseases (20). These abnormally expressed circRNAs have the potential to play a role in the onset and progression of diseases. Additionally, these molecules could potentially function as indicators for diagnosis or objectives for treatment of specific ailments. Here, we present a summary of the ongoing investigations regarding circRNAs in connection with bone-related diseases (**Figure 1**), including OS, osteoporosis (OP), osteonecrosis of the femoral head (OFNH), osteoarthritis (OA), and intervertebral disc degeneration (IDD).

## 2 Search strategy

This review is based on an extensive literature search conducted to compile the latest advancements in circular RNA (circRNA) research, specifically in the context of bone-related diseases. Articles were identified through a systematic search of electronic databases, including PubMed, Scopus, and Web of Science, using keywords such as “circular RNA,” “non-coding RNA,” “osteosarcoma,” “osteoporosis,” “osteonecrosis of the femoral head,” “osteoarthritis,” and “intervertebral disc degeneration.” Publications from 2000 to 2024 were considered, with priority given to peer-reviewed studies, high-impact journals, and recent advancements. Non-English publications, conference abstracts, and studies unrelated to the core focus of circRNA in orthopedic diseases were excluded. The titles and abstracts of identified articles were screened for relevance. Full-text articles were assessed to ensure alignment with the review’s objectives. Additional studies were included through manual screening of references in selected articles. The selection criteria focused on studies elucidating circRNA mechanisms, detection technologies, and their roles in orthopedic disease pathogenesis, diagnosis, and therapeutic targeting. Key information was extracted from the selected studies, including experimental methods, circRNA targets, disease contexts, and clinical implications.

## 3 Overview of circRNAs

### 3.1 Biogenesis of circRNAs

CircRNAs are primarily formed through back-splicing of mRNA precursor (pre-mRNA) (10) and are categorized into three groups based on their source sequences: exonic circRNAs (EcRNAs), intronic circRNAs (IcRNAs), and exon-intron circRNAs (EIcRNAs) (6). There are four main looping models reported: spliceosome-dependent lasso-driven circularization, intron reverse complementary pairing-driven circularization, RBPs-driven circularization, and tRNA precursor splicing pathway.

### 3.2 Biological characteristics of circRNAs

CircRNAs display the subsequent biological features: (1) Diversity: In the circBase database, in 2014, 140,000 circRNAs were incorporated for humans, surpassing the number of protein-coding genes by over 30,000 (21). (2) Stability: The absence of a polyadenylate tail structure prevents circRNAs from degradation by ribonucleic acid exonuclease (RNaseR) due to their characteristic closed-loop structure with covalently linked 3’ and 5’ ends (5, 22). (3) High species conservation: A recent study revealed that 72.6% of all circRNAs identified in humans, macaques, and mice were highly conserved throughout evolution (23). Furthermore, the DNA sequences encoding circRNAs are more conserved than the non-coding flanking sequences (24). Additionally, circRNAs demonstrate distinct tissue and cell specificity, rendering them promising biomarkers for diagnosing certain diseases (3, 17, 25).

### 3.3 Degradation of circRNAs

CircRNAs are relatively stable with a half-life of 18.8-23.7 hours, which is much higher than that of their linear transcripts (4.0-7.4 hours) (26). The degradation mechanism of circRNAs is not clear, and there are four reported modes of circRNA degradation. (1) MiRNA sequence-dependent AGO2-mediated degradation. For example, CDR1as/ciRS-7, through recruitment and complementary pairing with miR-671, causes AGO2 endonuclease cleavage to disrupt the closed-loop structure. Subsequently, the endonuclease further degrades the linear RNA (27). (2) Methylation modification of circRNAs (m6A) induces sequence-dependent degradation by endonucleases RNase P/MRP. Park et al. (28) found that circRNAs containing m6A methylation modification can recruit YTHDF2 and HRSP12 proteins. These proteins act as a bridge between RNase P/MRP and circRNA-specific sequences, facilitating circRNA degradation. (3) PKR-activated RNase L-mediated degradation. Liu et al. (29) found that the protein kinase PKR was abnormally activated during the course of systemic lupus erythematosus. Subsequently, the cytoplasmic endonuclease RNase L was activated and degraded circRNAs. (4) UPF1- and G3BP1-dependent degradation. UPF1 and G3BP1 degrade circRNAs by recognizing the higher-order structure of circRNAs and activating the helicase and endonuclease. This degradation mode is known as structure-mediated RNA degradation because the entire RNA structure is recognized, not just the nonlinear primary structure (30). (5) GW182-mediated degradation. Jia et al. (31) found that circRNAs are directed into specific degradation pathways by the evolutionarily conserved factor GW182.

## 4 Technology of circRNAs

Advances in technology have greatly facilitated circRNA research by enabling their detection, analysis, and functional investigation (32–35). This section compares key technologies in circRNA studies, emphasizing their respective strengths and limitations, as well as their scope of application.

### 4.1 Genome-wide analysis of circRNAs

Genome-wide detection methods, including RNA sequencing (RNA-seq) and microarrays, serve as primary tools for identifying circRNAs. RNA-seq provides high-resolution, large-scale data and identifies circRNA-specific backsplice junctions (BSJ) (36–38). Despite its power, the lack of a standardized bioinformatics algorithm requires the simultaneous use of multiple tools to ensure accuracy. In contrast, microarrays provide a faster and more sensitive alternative, especially with the use of RNase R pretreatment and antisense probes (39, 40). However, microarrays cannot detect novel circRNAs or quantify the relative ratios of circRNA to linear transcripts due to sample de-linearization.

### 4.2 Analysis of CircRNA site specificity

CircRNA-specific detection methods, such as quantitative reverse transcription PCR (RT-qPCR) and droplet digital PCR (ddPCR), allow for targeted and precise validation (41, 42). RT-qPCR is cost-effective and widely used, while ddPCR provides superior sensitivity and quantification accuracy, particularly in challenging samples, such as plasma (43–45). High-throughput alternatives, including NanoString nCounter, enable simultaneous detection of up to 800 circRNAs, making it a valuable tool for large-scale studies.

### 4.3 Visualization and localization of circRNAs

*In situ* hybridization (ISH) remains a cornerstone for visualizing circRNAs in cells and tissues. Advances, such as BaseScope (35), improve signal clarity and sensitivity by optimizing probe designs. Recently, CRISPR-Cas13-based techniques have emerged as promising tools for real-time circRNA tracking, enabling dynamic localization studies with fluorescent tagging systems (36–38).

### 4.4 Knockdown and overexpression of circRNAs

Knockdown and overexpression techniques are critical for understanding circRNA functions. RNA interference (RNAi) is widely employed for transient knockdown, while lentivirus-based approaches offer long-term suppression (46). Overexpression methods, such as plasmids with flanking sequences promoting circularization, provide efficient tools for gain-of-function studies. Adding specific RNA-binding protein (RBP) binding sites further enhances expression efficiency.

### 4.5 Knockout of circRNAs

Gene-editing technologies, such as CRISPR-Cas9, facilitate circRNA knockout by targeting critical flanking sequences or directly deleting coding exons (47, 48). These approaches require careful validation to avoid unintended effects on host gene expression. Knockout models, such as those for ciRS-7, demonstrate the utility of these methods in elucidating circRNA functions (49–51).

### 4.6 Comparison and applications

RNA-seq excels in exploring novel circRNAs, while microarrays are suggested for routine studies of known circRNAs. ddPCR and NanoString provide precise quantification, and NanoString is more applicable for high-throughput analyses. ISH and CRISPR-Cas13 enable spatial studies, while RNAi and gene editing support functional investigations. The selection of technique depends on study objectives, with genome-wide methods suited for discovery and targeted approaches ideal for validation and mechanistic investigations.

## 5 CircRNAs and orthopedic-related diseases

### 5.1 CircRNAs and OS

OS, also known as osteogenic sarcoma, is the most prevalent type of primary malignant bone tumor (44), usually found in the epiphysis of long tubular bones. This type of tumor is commonly diagnosed in individuals aged between 15 to 25 years old. In clinical practice, diagnosing and treating OS remains difficult because the cancer is highly resistant to chemotherapy and tends to metastasize to the lungs early on (45). In recent years, circRNAs and OS have been increasingly studied and reported in relation to OS cell proliferation, apoptosis, energy metabolism, angiogenesis, metastasis, chemotherapy sensitivity, and drug resistance.

#### 5.1.1 Expression profile of circRNAs in OS

Firstly, circRNAs exhibit differential expression at both the transcriptional level in OS tumor tissue samples and in commercial cell lines. Recent studies have highlighted specific circRNAs with aberrant expression patterns and functional roles in OS progression. For instance, circ_0001785 is markedly upregulated in OS tissues and has been implicated in promoting tumor proliferation and invasion through the miR-1200/STAT2 axis. Similarly, circ_0016347 facilitates OS progression by sponging miR-214-5p and regulating the EZH2/STAT3 signaling pathway. In contrast, circ_0001564 exhibits downregulated expression in OS and acts as a tumor suppressor by targeting the miR-29c-3p/CDC42 axis.

Comprehensive bioinformatics analyses have also elucidated the involvement of circRNAs in key signaling pathways, such as the PI3K/AKT and Wnt/β-catenin pathways, which are frequently dysregulated in OS. These findings underscore the pivotal role of circRNAs in OS pathogenesis and provide a basis for identifying novel diagnostic biomarkers and therapeutic targets.

Previous studies have further examined a large number of differentially expressed circRNAs through genome-wide analysis using OS patients and normal human tissues or body fluids, such as serum. These studies have predicted target genes and signaling pathways using bioinformatics techniques to construct complex regulatory networks, providing important references for subsequent mechanistic studies of the disease. For instance, Xi et al. (52) compared OS samples from three patients with adjacent tissues using a microarray chip. They discovered that a total of 259 circRNAs exhibited differential expression patterns; among these, 132 circRNAs showed upregulation, while 127 circRNAs were downregulated. Similarly, Chen et al. (53) found that 8 circRNAs were upregulated and 102 were downregulated in the OS group when analyzing OS cell lines (MG63, Saos-2, and U2OS) and the control group (hFOB osteoblast cell line). By integrating these insights, the current understanding of circRNAs in OS continues to evolve, providing valuable direction for future mechanistic and clinical studies.

#### 5.1.2 CircRNAs as biomarkers in OS

The structure of circRNAs makes them biologically stable, tissue-specific, and conserved across species. In addition to tumor tissue cells, circRNAs are also widely expressed in human plasma, plasma exosomes, extracellular vesicles, and exosomes. Due to these unique biological properties, differentially expressed circRNAs offer significant advantages in clinical practice as biomarkers for OS diagnosis, clinical staging, and prognosis assessment (refer to **Table 1**). For example, Lou et al. (46) found that circUSP34 had upregulated expression in both 143B and KHOS cells and OS tissues. Zhu et al. (47) found that circPVT1 was upregulated in OS tissues by analyzing 80 pairs of OS and adjacent tissues. The area under the receiver operating characteristic curve (ROC) of circPVT1 as a diagnostic marker for OS was 0.871, while the AUC of the conventional tumor marker alkaline phosphatase (ALP) was 0.673. This suggests that circPVT1 shows promise as a diagnostic marker for OS. In terms of prognostic evaluation, the expression of circPVT1 in OS tissues is related to overall survival, with a significant negative correlation observed. According to a study by Zhu et al. (48), r high expression levels of circ_0081001 in OS tissues and serum can serve as a potential diagnostic marker for OS, the AUC of the diagnostic experiment was 0.898. This value indicated a higher diagnostic efficiency than ALP (AUC = 0.673) and lactate dehydrogenase (LDH) (AUC = 0.80). In the small prospective preliminary clinical trial, monitoring the dynamic changes of serum Circ_0081001 can timely and accurately reflect the chemotherapy resistance of OS patients or disease progression in lung metastases, confirming that it has considerable applications in the clinical monitoring of disease progression in OS. At the level of clinical prognostic evaluation, Zhang et al. (49) collected and summarized the clinical data of abnormally expressed circRNAs and 1979 OS patients in 31 studies using big data. In their study, it was also confirmed that abnormally expressed circRNAs showed a strong correlation with various clinical parameters (such as Enneking stage, tumor size, and distant metastases) and clinical prognosis (such as overall survival and progression-free survival) of OS patients.

**Table 1:** Studies on circRNA molecules as diagnostic markers for osteosarcoma.

CircRNA	Sample type	Samples		Expression	AUC	Date	Reference
		Case	Control				
circEMB circROCK1 circLRP6 circ_0010220 circ_001422 circUBAP2	OS and ANT OS and ANT OS and ANT serum OS and ANT OS and ANT	53 50 50 19 55 42	53 50 50 19 55 42	up down up down up up	0.703 0.9048 0.690 0.803 0.752 0.7664	2023 2022 2022 2022 2021 2021	(50) (17) (51) (54) (55) (56)
circHIPK3	OS and chondrosarcoma	12	12	up	0.875	2021	(57)
circ_0056285	serum	35	35	up	0.778	2021	(58)
circ_0000190	serum	60	60	down	0.889	2020	(59)
circ_ 0003074	plasma	40	30	up	0.93	2020	(60)
circ-LARP4	OS and ANT	72	72	down	0.829	2020	(61)
circCNST	OS and ANT	126	126	up	0.63	2020	(62)
circ_0000885	serum	30	25	up	0.783	2019	(63)
circ_HIPK3	serum	50	20	down	0.783	2018	(64)
CDR1as	OS and ANT	38	18	up	0.857	2018	(65)
circ_0008717	OS and ANT	45	45	up	0.782	2018	(66)
circPVT1	serum	50	20	up	0.871	2018	(47)
circ_0081001	serum	50	20	up	0.898	2018	(48)

*OS, osteosarcoma; ANT, adjacent normal tissue.

#### 5.1.3 Involvement of circRNAs in the development of OS

During OS development, circRNAs primarily act as “molecular sponges” that compete with microRNAs (miRNAs) for binding to mRNA-binding sites. They regulate downstream genes post-transcription, influencing biological processes such as tumor cell proliferation, invasion, apoptosis, and energy metabolism. CircRNAs with increased expression in OS tissues are typically believed to promote cancer. The research conducted by Yu et al. (67) revealed an increase in circFIRRE expression in both OS tissues and cell lines. Mechanistic studies revealed that it could bind to the miRNAs miR-486-3p and miR-1225-5p through a sponge effect, leading to the upregulation of the downstream oncogene LUZP1. Functional experiments demonstrated that it could enhance OS cell proliferation, invasion, and migration as evidenced by CCK-8, transwell assay, and wound healing assay, thereby facilitating the progression of OS. High expression of circMYO10 in OS tissues increased the transcriptional activity of the β-catenin/LEF1 complex by regulating miR-370-3p. This also promoted the proliferation and invasion of OS cells (68). CircRNAs can also influence tumor progression by affecting tumor energy metabolism pathways. Shen et al. (69) found that high expression of circECE1 in OS tissues inhibited c-Myc ubiquitination degradation, thereby activating the Warburg effect. This enhanced anaerobic glycolysis to supply energy to the tumor and promoted the development of OS.

In contrast to the high expression of oncogenes, the relatively low expression of circRNAs in tumor tissues typically inhibits OS progression. CircITCH is expressed at low levels in tumor tissues from OS patients. It indirectly inhibits the PTEN/PI3K/AKT and SP-1 pathways by adsorbing miR-22, which functionally inhibits the proliferation of OS cells (MG63, Saos-2), promotes apoptosis, and ultimately plays a tumor suppressor role (70). Not only can circRNAs inhibit tumor progression *in situ*, but they can also play a tumor-suppressive role in adjacent normal tissues in OS. For instance, low-level expression of hsa_circ_0000190 in the serum of OS patients also occurs in normal bone tissue (osteoblast cell line hFOB) and is secreted by extracellular vesicles (EVs) to facilitate intercellular communication. This process ultimately targets OS cells to hinder their proliferation and invasion (60).

The involvement of circRNAs in OS is primarily mediated by their function as molecular sponges, modulating miRNA activity, thereby regulating downstream gene expression. This mechanism influences critical cellular processes, such as tumor cell proliferation, invasion, and energy metabolism. For instance, the sponge-like activity of circRNAs like circFIRRE and circMYO10 promotes oncogenic pathways, whereas the tumor-suppressive effects of circITCH are achieved through inhibition of pathways like PTEN/PI3K/AKT. These mechanisms highlight the dual role of circRNAs, either facilitating or inhibiting OS progression, depending on their expression patterns.

#### 5.1.4 Involvement of circRNAs in OS lung metastasis

Nowadays, the prognosis of OS has significantly improved with multidisciplinary treatment. However, distant metastases remain a crucial factor affecting long-term survival, with pulmonary metastases being the most common. There are numerous articles discussing the study of circRNAs and OS lung metastases. For example, Yan et al. (71) found that high expression of circPVT1 in OS can regulate FOXC2 post-transcriptionally through a ceRNA mechanism, ultimately promoting FOXC2 protein-mediated lung metastasis in OS. In the progression of OS, CAMP-responsive element-binding protein 3 (CREB3) plays a crucial role as a driver gene. A study constructed by Wu et al. (72) revealed that circTADA2A, exhibiting elevated levels in OS tissues, stimulated the proliferation and invasion of tumor cells by activating the miR-203a-3p/CREB3 pathway. At the same time, *in vivo* imaging (IVIS) and immunohistochemistry experiments in nude mouse models demonstrated that it significantly promotes OS lung metastasis. Vascular endothelial growth factor (VEGF) has been shown to be a key factor in lung metastasis in OS, and its targeted drugs, apatinib and sorafenib, have been shown to inhibit lung metastasis in OS. Ji et al. (73) found that a relatively high expression of circ_001621 can upregulate the expression level of VEGF through the ceRNA mechanism and promote the malignant biological behavior of OS cells. Animal experiments confirmed that high expression of VEGF promoted OS lung and liver metastasis. Circ-0000658, which is poorly expressed in OS tissues and cell lines, can inhibit OS lung metastasis through the miR-1227/interferon regulatory factor-2 (IRF2) regulatory axis. Therefore, these abnormally expressed circRNAs can be used as potential therapeutic targets for OS lung metastasis.

#### 5.1.5 Involvement of circRNAs in the mechanism of drug resistance in OS

OSs do not respond to radiotherapy, and the survival rate of patients with simple surgical resection is low. Currently, the standard treatment approach for OS typically involves a combination of preoperative neoadjuvant chemotherapy, surgery, and postoperative adjuvant chemotherapy. The 5-year survival rate of OS patients is 50-80%. However, with prolonged survival, chemotherapy resistance has become a significant clinical challenge for some patients. Several studies have demonstrated the direct relationship between circRNAs and resistance to OS and have described the relevant mechanisms (**Table 2**). For instance, when comparing three groups of doxorubicin-, cisplatin-, and methotrexate-multidrug-resistant cell lines (MG63R) with chemotherapy-sensitive cell lines (MG63), Zhu and colleagues (85) detected a collective of 80 dissimilarly expressed circRNAs, among which 57 were elevated and 23 were reduced in the resistant cells. Another investigation by Zhu and team (47) proved that circPVT1 was notably amplified in the cancerous tissues, blood samples, and drug-resistant cell lines (MG63R, U2OSR) of three OS patients. This overexpression was found to be correlated with poor prognosis for the patients. The researchers found that the cell viability of OS-resistant cell lines and their IC50 values for chemotherapeutic agents (doxorubicin, cisplatin) were significantly reduced by siRNA-specific knockdown of circPVT1. This suggests that circPVT1 can decrease the chemoresistance of OS. This process is mediated by the downregulation of ATP-binding cassette subfamily B member 1 (ABCB1) to regulate tumor resistance. Li et al. (87)investigated the highly expressed circ_0000073 in OS using the GEO database and demonstrated that circ_0000073 promotes methotrexate resistance by upregulating the oncogene NRAS.

**Table 2:** Studies on the correlation between circRNAs and chemotherapy resistance of OS.

CircRNA	Drug	Drug-resistant cell line	Expression	Mechanism	Date	Reference
circ-CHI3L1.2	cisplatin	MG63, Saos-2	up	miR-340-5p/LPAATβ regulatory axis	2021	(74)
circ_103801	cisplatin	MG63	up	Upregulate MRP1 and P-gp expression	2021	(75)
circUBAP2	cisplatin	U2OS, Saos-2	up	miR-506-3p/SEMA6D regulatory axis	2020	(76)
circTADA2A	cisplatin	U2OS, MG63	up	miR-129-5p/TRPS1/YAPS regulatory axis	2020	(77)
circ_001569	cisplatin	U2OS, MG63	up	Activate Wnt/β-catenin signal pathway	2018	(78)
circ_0001721	adriamycin	KHOS, MG63	up	miR-758/TCF4 regulatory axis	2021	(79)
circPVT1	adriamycin	KHOS, U2OS	up	miR-137/TRIAP1 regulatory axis	2021	(80)
circPRDM2	adriamycin	KHOS, MG63	up	miR-760/EZH2 regulatory axis	2021	(81)
circITCH	adriamycin	KHOS, MG63	down	miR-524/RASSF6 regulatory axis	2021	(82)
CircSAMD4A	adriamycin	HOS, U2OS	up	miR-218-5p/KLF8 regulatory axis	2020	(83)
circ_0003496	adriamycin	KHOS, MG63	up	miR-370/KLF12 regulatory axis	2020	(84)
circ_0004674 circ_0081001	adriamycin adriamycin	KHOS, MG63, U2OS KHOS, MG63, U2OS	up up	miR-490-3p/ABCC2 and miR-1254/EGFR regulatory axis /	2018 2018	(85) (48)
circ_0081001	methotrexate	HOS, U2OS	up	miR-494-3p/TGM2 regulatory axis	2021	(86)
circ_0000073 circ‐LARP4 circPVT1	methotrexate cisplatin, Adriamycin cisplatin, Adriamycin	U2OS, MG63 MG63, Saos-2 KHOS, MG63, U2OS	up down up	Sponge miR-145-5p and miR-151-3p, regulate downstream NRAS Sponge miR-424 Upregulate ABCB1 expression	2020 2019 2018	(87) (61) (47)

*Expression: the expression in chemotherapy-resistant osteosarcoma; OS, osteosarcoma.

### 5.2 circRNAs and OP

OP is a systemic disease affecting the skeletal system, marked by reduced bone mass and deterioration in bone structure, leading to bone fragility and increased fracture risk (88). According to statistics, the prevalence of OP among middle-aged and elderly men in China was 20.73%, while it was 38.05% among middle-aged and elderly women (89). It is expected that the number of OP patients in China will increase to 5.99 million by 2050, incurring medical costs of 174.5 billion yuan (90). The development of OP is caused by an imbalance in bone formation by osteoblasts and bone resorption by osteoclasts. Decreased osteoblast production and increased osteoclast activity in response to various pathogenic stimuli lead to continuous bone loss, eventually causing the development of OP. There are also a large number of reports on circRNAs involved in the imbalance between osteogenesis and osteoclasts.

OP arises from an imbalance in bone remodeling, characterized by reduced osteoblast activity and heightened osteoclast-mediated bone resorption. CircRNAs contribute to this imbalance by regulating key molecular pathways. For instance, circRNAs, such as circRNA_0048211 and circRNA_0016624, promote osteogenesis by modulating BMP signaling, while circRNA_28313 facilitates osteoclast differentiation via the miR-195a/CSF1 axis. These findings highlight the role of circRNAs in orchestrating the complex interplay between osteoblast and osteoclast activity, ultimately driving the development of OP.

#### 5.2.1 Expression profile of circRNAs in OP

In a recent study, Yu et al. (91) identified 221 circRNAs that were significantly upregulated and 176 circRNAs that were significantly downregulated in OP patients by comparing six pairs of OP patients with normal human serum. The five circRNAs with the most significant expression differences were circ_0016624, circ_0134944, circ_0057340, circ_0062466, and circ_0116994. Zhi et al. (92) compared three pairs of OP patients with normal human serum exosome samples using microarray analysis. They found that 589 circRNAs were differentially expressed in the two groups. Among these, 376 circRNAs were relatively strongly expressed, while 213 were relatively weakly expressed compared to the normal group. Shen et al. (93) compared 20 pairs of osteoporotic and normal bone tissues using microarray chip analysis. They identified 2645 circRNAs with relatively high expression and 2327 with relatively low expression at the same critical value (fold change > 2). These circRNAs with significant differences in expression can be further investigated and are expected to become new diagnostic and therapeutic biomarkers for OP. Zhang et al. (94) identified 398 differentially expressed circRNAs by sequencing the entire transcriptome of peripheral blood samples from three pairs of male OP patients and individuals over 60 years old. They also established a circRNA-miRNA-mRNA regulatory network using differentially expressed miRNAs and mRNAs. This network offers a valuable foundation for future studies on the regulatory mechanisms of OP.

#### 5.2.2 CircRNAs as biomarkers in OP

In the current study, many circRNAs have been identified as potential biomarkers with significant promise for the early diagnosis and assessment of OP. Guan et al. (95) studied 28 pairs of postmenopausal osteoporosis (PMOP) and normal peripheral blood samples from women. They found that the expression of circ_0021739 was upregulated in PMOP patient samples. The sensitivity as a diagnostic marker for PMOP was 100%, specificity was 42.9%, and the AUC was 0.849. Bone loss as an early manifestation of OP is reflected by a decrease in bone volume per unit of bone. Xiang et al. (96) reported that circ 0001445, with low expression in the plasma of PMOP patients has high diagnostic efficacy in diagnosing osteopenia and OP. The sensitivity and specificity for diagnosing bone loss were 70.67% and 77.78%, respectively, with an AUC of 0.8115. For diagnosing OP, the sensitivity and specificity were 97.62% and 82.22%, respectively, with an AUC of 0.9589. In the differential diagnosis of bone loss and OP, the sensitivity and specificity were 78.57% and 69.33%, respectively, with an AUC of 0.8298. In the serum exosomes of OP patients, Zhi et al. (92) found that circ_0006859 was also identified as a reliable biomarker for diagnosing osteopenia and OP. The area under the AUCs for osteopenia, OP, and their differential diagnosis were 0.913, 0.8974, and 0.8873, respectively. The significance and value of circRNAs in the diagnosis and prognosis assessment of OP are immense. In subsequent studies, the simultaneous detection of multiple circRNAs at multiple sites can be considered, potentially resulting in increased diagnostic efficiency.

#### 5.2.3 CircRNAs and OP

Osteogenesis is primarily mediated by osteoblast-related cells, specifically osteoblasts, which are mainly differentiated from bone mesenchymal stem cells (BMSCs). Bone morphogenetic protein (BMP) can be used as an inducer to stimulate BMSCs to differentiate into osteoblasts. Among BMPs, BMP-2 is the most potent factor capable of independently inducing osteogenesis. Qiao et al. (97) found that circRNA_0048211 was downregulated in the bone marrow of PMOP patients. It could act as an adsorption sponge for miRNA-93-5p, upregulating BMP2 levels. This process promoted osteogenic differentiation of BMSCs, thereby alleviating the progression of PMOP. Yu et al. (91) found that the expression of circRNA_0016624 and BMP2 was relatively downregulated in PMOP patients based on serum sequencing. CircRNA_0016624 was shown to indirectly upregulate BMP2 expression by sponging miR98. Alizarin red staining demonstrated that overexpression of circRNA_0016624 could enhance osteogenic function.

Melatonin, a hormone that regulates biological circadian rhythms, has also been shown to be involved in bone metabolism. Clinical studies suggest that melatonin helps increase bone mass in postmenopausal women (98, 99). Wang et al. (100) demonstrated that melatonin can promote osteogenic differentiation of BMSCs through ALP staining and alizarin red staining. Sequencing analysis of melatonin-treated and untreated BMSCs revealed that melatonin treatment significantly reduced the expression of circ_0003865 in BMSCs. Functional experiments showed that melatonin regulates the expression of the downstream gene GAS1 by inhibiting the expression of circ_0003865, thereby promoting osteogenic differentiation.

Osteogenic-vascular coupling is a new theory proposed in recent years to explain the pathogenesis of osteoporosis. Ji et al. (101) reported that the expression of circ_0006215 was decreased in BMSCs from OP patients. This decrease not only promotes osteogenic differentiation of BMSCs by regulating the RUNX2 gene but also upregulates the expression of VEGF in BMSCs to promote neovascularization. The osteogenic/angiogenic capabilities of circ_0006215 make it one of the potential therapeutic targets for OP patients. In addition to the aforementioned osteoblast-related studies, numerous studies have confirmed that circRNAs influence the OP process by regulating osteoblast proliferation and differentiation (**Table 3**).

**Table 3:** Role of circRNAs in regulating osteoblast/osteoclast differentiation in osteoporosis.

CircRNA	Osteogenesis/osteoclastogenesis	Expression	Mechanism	Date	Reference
circEIF4B circFam190a circ_0114581 circStag1 circ_0001485 circRNA−23525	osteogenesis osteoclastogenesis osteogenesis osteogenesis osteogenesis osteogenesis	up up up down up up	Phytic acid/circEIF4B/miR-186-5p/ITGA5 regulatory axis FUS/circFam190a/HSP90β/AKT1 regulatory axis miR-155-5p/HNRNPA3 regulatory axis circStag1/HuR regulatory axis, activite Wnt/β-catenin signaling pathway TGFβ-BMP signaling pathway miR-30a-3p/RUNX2 regulatory axis	2024 2023 2023 2022 2022 2021	(102) (103) (104) (105) (106) (107)
circ_0062582	osteogenesis	up	miR-145/CBFB	2021	(108)
circ_AFF4	osteogenesis	up	miR-135a-5p//FNDC5/Irisin regulatory axis	2021	(109)
circ_0006859	osteogenesis	up	miR-431-5p/ROCK1 regulatory axis	2021	(92)
circ_0006215	osteogenesis	down	Sponge miR-942-5p, regulate downstream RUNX2和VEGF	2021	(101)
circ_0003865	osteogenesis	down	melatonin/circ_0003865/miR-3653-3p/GAS1 regulatory axis	2021	(100)
circ_0076690	osteogenesis	down	Sponge miR-152	2020	(110)
circ_0024097	osteogenesis	up	miR-376b-3p/YAP1 regulatory axis, activate Wnt/β-catenin signaling pathway	2020	(111)
circ_0026827	osteogenesis	up	Sponge miR-188-3p, regulate downstream Beclin1 and RUNX1	2020	(112)
circRNA_0048211	osteogenesis	down	miRNA-93-5p/BMP2	2020	(97)
circ_0076906	osteogenesis	down	miR-1305/OGN regulatory axis	2020	(113)
circ_0011269	osteogenesis	down	miR‐122/RUNX2 regulatory axis	2020	(114)
circFOXP1	osteogenesis	down	miR-33a-5p/FOXP1 regulatory axis	2020	(93)
circ_0074834	osteogenesis	down	Sponge miRNA-942-5p, regulate downstream ZEB1和VEGF	2019	(115)
circRNA_33287	osteogenesis	up	miR-214-3p/Runx3 regulatory axis	2019	(116)
circRNA_0006393	osteogenesis	down	miR−145−5p and FOXO1 regulatory axis	2019	(117)
circ-VANGL1	osteogenesis	down	miRNA-217/RUNX2 regulatory axis	2019	(118)
circIGSF11	osteogenesis	up	Sponge miR-199b-5p	2019	(119)
circRUNX2	osteogenesis	down	miR‐203/RUNX2 regulatory axis	2018	(120)
circRNA_0007059	osteoclastogenesis	up	miRNA‐378/BMP‐2 regulatory axis	2021	(121)
circ_0021739	osteoclastogenesis	down	Sponge miR-502-5p	2021	(95)
circRNA_009934	osteoclastogenesis	up	miR-5107/TRAF6 regulatory axis	2020	(122)
circRNA_28313	osteoclastogenesis	up	miR-195a/CSF1 regulatory axis	2019	(123)
circ_0002922	osteoclastogenesis	down	miR-181b-5p/MAP2K1 regulatory axis	2020	(124)
circ_0007710	osteoclastogenesis	down	miR-197-3p/MAPK1;miR-20a-5p/MAPK9 regulatory axis	2020	(124)
circHmbox1	osteogenesis, osteoclastogenesis	down	mIR-1247-5p/TNF-αregulatory axis	2020	(125)

#### 5.2.4 circRNAs and OP bone resorption

Osteoclasts are the only cells in the human body that have the function of bone resorption, maintaining the balance of bone metabolism with osteoblasts under physiological conditions. Osteoclasts differentiate from bone marrow-derived macrophages (BMM) (126). In this process, macrophage colony-stimulating factor (M-CSF) promotes the proliferation of osteoclast precursors, while the differentiation of osteoclast precursors into mature osteoclasts is facilitated by the receptor activator for nuclear factor-κB ligand (RANKL). Chen et al. (123) found that circRNA_28313 expression increased significantly during the differentiation of BMMs into osteoclasts induced by RANKL + M-CSF. After the expression of circRNA_28313 was reduced by RNA interference, the differentiation of BMMs into osteoclasts and bone resorption were significantly inhibited in ovariectomized (OVX) mice. Mechanism studies have shown that circRNA_28313 can regulate osteoclast differentiation via the miR-195a/CSF1 signaling axis, thereby affecting bone resorption during the course of OP. Liu et al. (121) found that the expression of circ_0007059 was significantly higher than that of normal controls by sequencing PMOP patients and normal samples. TRAP staining was used to verify that circ_0007059 could inhibit the differentiation of BMSCs into osteoclasts. Finally, bioinformatics analysis and dual luciferase reporter gene experiments confirmed that circ_0007059 regulates osteoclast formation through the circ_0007059/miR-378/BMP-2 axis. Related studies that report the impact of circRNAs on OP through their influence on osteoclasts are summarized in **Table 3**.

### 5.3 CircRNAs and OFNH

OFNH, referred to as avascular necrosis of the femoral head, is a degenerative condition that may lead to the continuous collapse of the femoral head, deformity, degenerative arthritis, and dysfunction (127, 128). Steroid-associated OFNH (SONFH) is most prevalent in China, with approximately 150,000 to 200,000 new cases reported annually (129). The exact pathogenesis of ONFH remains unclear, and numerous studies have explored the role of circRNAs in its development. For instance, Yao et al. (130) examined three pairs of femoral head and normal tissues from SONFH patients using a microarray chip and identified 433 upregulated circRNAs and 214 downregulated circRNAs. Jiao et al. (131) discovered 74 differentially expressed circRNAs and 121 mRNAs in ONFH tissues through transcriptome sequencing analysis. This analysis included three cases of subchondral bone in ONFH patients and three cases of hip replacement patients. Subsequently, circ_0001187 and circ_0008928, which showed the highest upregulation in sequencing, were selected and confirmed to be highly expressed in ONFH tissues by RT-qPCR. These differentially expressed circRNAs may play a role in the formation and progression of ONFH. Furthermore, Jiang et al. (132) observed a significant upregulation of CDR1as expression in necrotic sites and plasma of 99 ONFH patients. CDR1as in plasma demonstrates good diagnostic efficacy as an early and intermediate diagnostic marker for ONFH. The AUC is 0.695 for the diagnosis of international ONFH stage (ARCO) 1/2, and the AUC is 0.635 for the diagnosis of ARCO stage 3/4. The dysregulation of circRNAs in ONFH suggests their potential role as both biomarkers for early diagnosis and targets for therapeutic interventions. However, the exact pathways through which circRNAs mediate osteonecrosis progression remain underexplored and warrant further study.

The pathogenesis of OFNH involves disrupted vascularization, oxidative stress, and impaired osteogenesis. CircRNAs, such as CDR1as, influence adipogenic differentiation by regulating the WNT5B gene, exacerbating the condition, while circHIPK3 counters oxidative damage to osteoblasts, mitigating disease progression. These findings indicate that circRNAs serve as both contributors and potential therapeutic targets in OFNH through their regulatory roles in cellular differentiation and stress responses.

Studies have also demonstrated the crucial role of BMSCs in the management of ONFH illness. Overconsumption of hormones may boost the adipogenic differentiation of BMSCs and impair their ability to differentiate into osteogenic cells (133). Feng et al. (134) found that circHGF suppressed the proliferation and osteogenic differentiation of BMSCs isolated from 10 patients with steroid-induced ONFH by interacting with miR-25-3p to modulate SMAD7. Xiang et al. (135) isolated BMSCs from seven patients with ONFH and found that the proliferation activity was significantly decreased, the apoptosis rate was increased, and bone formation was decreased. However, adipogenic differentiation was increased, indicating the involvement of BMSCs in the disease process of ONFH. Sequencing and cell function experiments showed that circ_0000219 and circ_0005936 may be involved in the regulation of ONFH by mediating the functional changes of BMSCs. Chen et al. (133) confirmed that the star molecule CDR1as was upregulated in SONFH. The WNT5B gene was regulated by the sponge adsorption of miR-7-5, promoting the adipogenic differentiation of BMSCs and inhibiting osteogenic differentiation. In ONFH disease, reactive oxygen species (ROS) can exacerbate disease progression by damaging osteoblasts and osteocytes. Liang et al. (136) found that circHIPK3, which is expressed at low levels in ONFH tissues, can reduce apoptosis by enhancing osteoblast activity to counteract the oxidative damage caused by ROS to osteoblasts. The biological mechanism of circHIPK3 in counteracting ROS damage and ONFH should be further explored. It is anticipated that circHIPK3 could potentially serve as a molecular target for disease treatment. Future studies will concentrate on uncovering the interplay between circRNAs, ROS generation, and the balance between adipogenic and osteogenic differentiation in BMSCs to provide a clearer picture of their role in OFNH.

### 5.4 CircRNAs and OA

**Figure 2:**
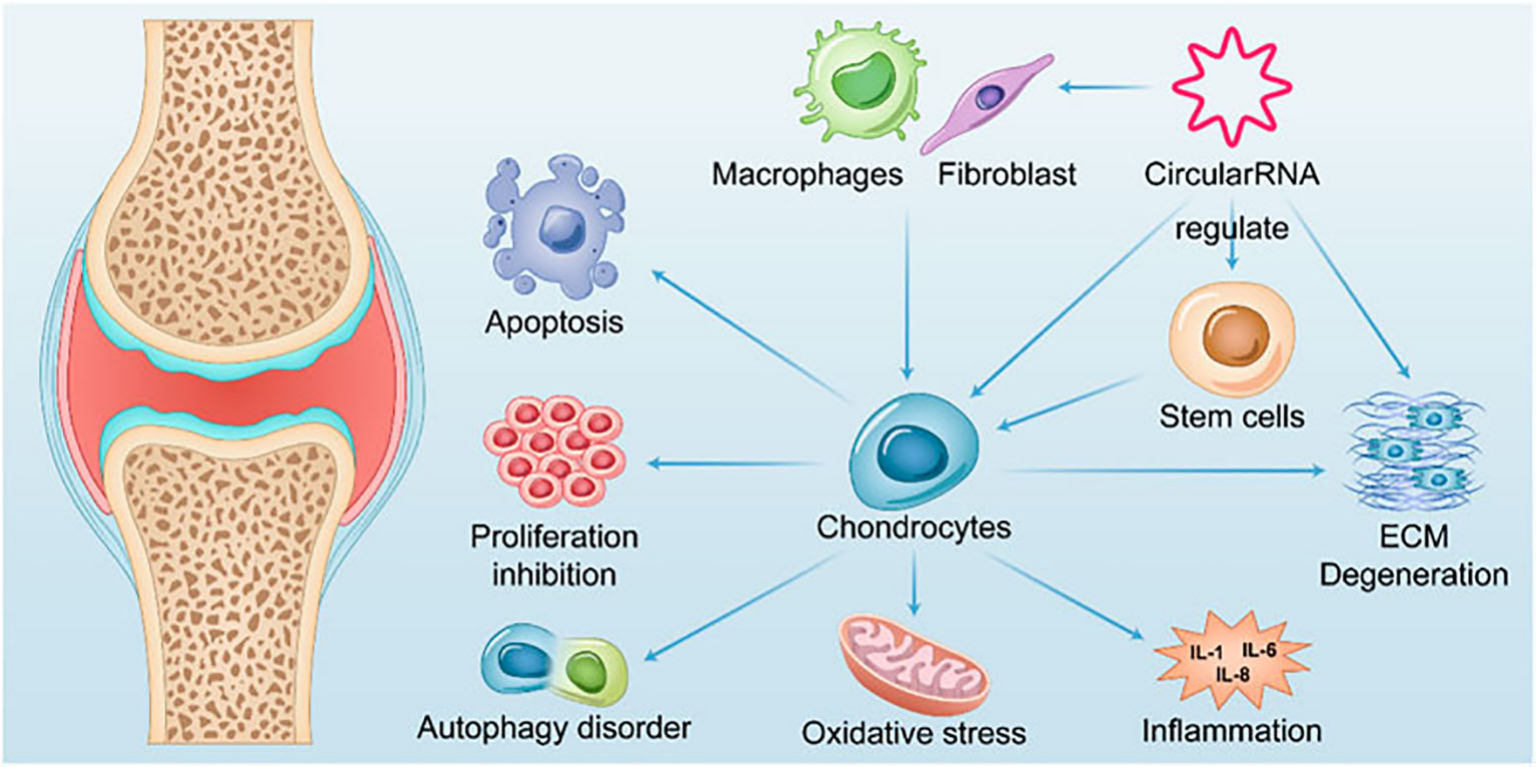
Summary of the role of CircRNAs in OA.

OA is the most common degenerative joint disease (137) and is pathologically characterized by degenerative necrosis of articular cartilage and reactive hyperplasia of the joint margins and subchondral bone. Arthroplasty is the accepted treatment for advanced stages of the disease, but functional recovery and the longevity of prostheses remain unsatisfactory (138). Due to the lack of therapeutic interventions, the current clinical focus is mainly on the prevention and early treatment of OA. The identification of different disease-specific biomarkers has offered fresh insights into diagnosing and treating OA at an early stage. The role of circRNAs in OA is illustrated in **Figure 2**.

Li et al. (139) investigated 42 differentially expressed circRNAs using high-throughput sequencing of articular cartilage tissue from OA patients and normal controls. Wang et al. (140) studied 1627 differentially expressed circRNAs in cartilage from five pairs of OA and Kaschin-Beck disease (KBD). The most down-regulated circRNA_0020014 was identified, which could be used to differentiate between OA and KBD with an AUC of 0.6415. This circRNA could potentially serve as a biomarker for the distinct diagnosis of OA. In a study on knee OA synovitis, Wang et al. (141) found that the expression of circ_0005526 (circ_RUNX2) was significantly increased in the serum of 60 patients with OA by screening circRNAs derived from RUNX2. When circ_RUNX2 was used as a diagnostic marker for OA, the sensitivity and specificity were 0.78% and 77%, respectively, and the AUC was 0.82, indicating its great potential as a diagnostic marker for OA.

**Figure 3:**
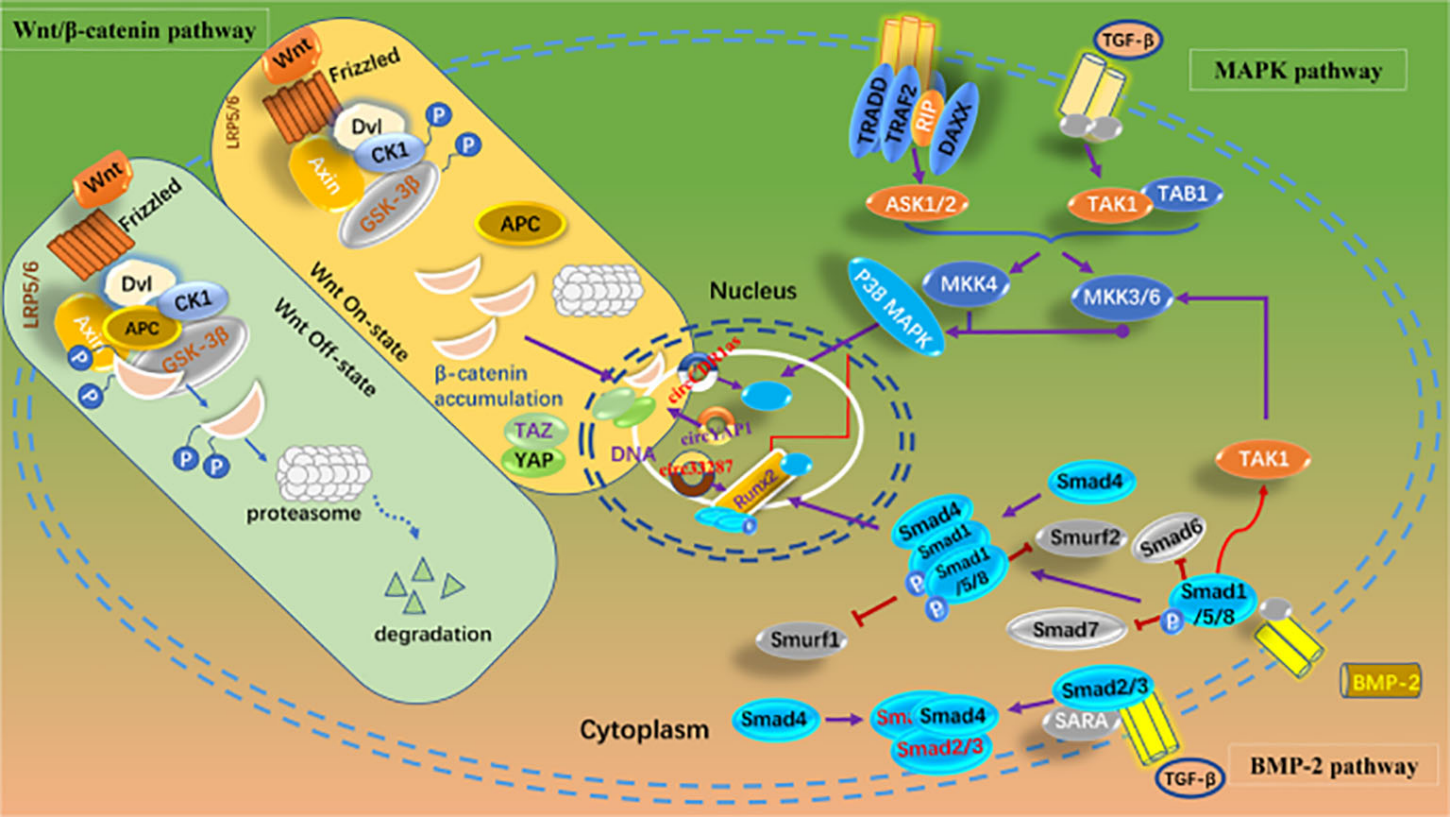
Signaling pathways regulated by circRNAs in OA.

Mediating chondrocyte proliferation, apoptosis, and autophagy; inhibiting extracellular matrix (ECM) degradation; and regulating the expression of signaling molecules in inflammation-related pathways are the primary theoretical underpinnings of current research on OA therapy. Liang et al. (142) found that the expression of circGNB1 increased in chondrocytes under inflammatory conditions. The inhibition of circGNB1 resulted in a significant reduction in intracellular ROS and broad inhibition of ECM degradation. Xu et al. found that circCREBBP is upregulated in osteoarthritic tissues and chondrocytes isolated from the cartilage of OA patients (143). AAV-sh-circCrebbp intra-articular injection alleviated the degenerative changes in the cartilage of mice with DMM-induced OA, resulting in lower Osteoarthritis Research Society International (OARSI) scores for the knee joints compared to those in the control group. Mechanistically, circCREBBP promotes the progression of OA by sponging miR-1208 in the TGFβ2-Smad1/5 pathway. This suggests that targeting circCREBBP to promote OA progression could potentially become a new target for precision therapy in OA. Despite these findings, the precise regulatory networks through which circRNAs modulate inflammatory cytokines and ECM catabolism in OA remain insufficiently characterized. Understanding these pathways can provide directions for targeted therapies that mitigate joint degradation. The signaling pathways regulated by circRNAs in OA are presented in **Figure 3**.

Articular cartilage is composed of ECM and chondrocytes. The ECM is in a dynamic equilibrium of continuous synthesis and degradation under physiological conditions. However, under pathological conditions, an imbalance between ECM synthesis and catabolism leads to OA (144). In the ECM degradation system, matrix metalloproteinases (MMPs) are the most important. Tang et al. (145) reported that circNFKB1, which is highly expressed in the cartilage of OA patients, can inhibit the expression of ECM anabolism genes ACAN and COL2A1 by sustaining the activation of the NF-κB signaling pathway and upregulate matrix metalloproteinase-13 (MMP-13) to promote ECM degradation. CircARPC1B (146) has also been shown to prevent ECM degradation by downregulating MMP-13 expression during OA. Gong et al. (147) found that the high expression of CircZSWIM6 in the cartilage of OA patients disrupted the balance between ECM catabolism and anabolism, leading to promoted ECM degradation. Intra-articular injection of adenoviruses overexpressing CircZSWIM6 exacerbated OA in mouse models. Endochondral inflammation in OA patients can lead to cartilage degradation and worsen the disease. Traditional inflammatory factors mainly include tumor necrosis factor-α (TNF-α), interleukin-1β (IL-1β), interleukin-6 (IL-6), transforming growth factor-β (TGF-β), etc. Liang et al. (142) found that circGNB1 was highly expressed in cartilage tissue and in chondrocytes induced by IL-1β or TNF-α in 30 OA patients. CircGNB1 regulates the downstream gene RNF219 by sponging miR-4152-3p, thereby inhibiting IL-1β-induced chondrocyte ECM degradation. CircIRAK3, which is highly expressed in OA, can also promote the expression of IL-1β, TNF-α, and IL-6 by binding to HNRNP U, leading to ECM degradation (148).

In OA, circRNAs regulate chondrocyte activity, ECM integrity, and inflammatory signaling. Pro-inflammatory circRNAs, such as circNFKB1 and circARPC1B, sustain NF-κB activation and MMP-13 expression, accelerating ECM degradation. Conversely, circRNAs, such as circCREBBP, mitigate cartilage degeneration by inhibiting miRNA pathways linked to the TGFβ2-Smad1/5 axis. These mechanisms illuminate the multifaceted roles of circRNAs in driving OA progression and inflammation.

### 5.5 CircRNAs and IDD

IDD is a common degenerative spinal condition characterized by loss of disc structure and function. Studies have identified aberrant expression of circRNAs in degenerated disc tissues, suggesting their involvement in cellular senescence, extracellular matrix metabolism, and inflammation. For example, circ_0000129 was found to regulate nucleus pulposus cell apoptosis via the miR-296-5p/PRKACB axis, highlighting its role in IDD pathogenesis. Although the evidence indicates the potential of circRNAs as therapeutic targets for IDD, further research is needed to delineate the mechanisms underlying their contribution to disc degeneration and regeneration. IDD is mainly marked by an irreversible halt in the growth of nucleus pulposus cells (NPCs) (149), which eventually results in diminished intervertebral height, endplate hardening, and a decline in the ECM (149). In recent years, abnormalities in nucleus pulposus cell function, including decreased proliferative activity, an imbalance between ECM production and degradation, and cytokine secretion, have been found to be closely related to the development and progression of IDD (150).

Wang et al. (151) isolated and identified nucleus pulposus cells from IDD patient tissues. They conducted a microarray analysis comparing these cells with nucleus pulposus cells from vertebral fractures. The study revealed that 3570 circRNAs were upregulated and 3724 were downregulated in the IDD patient tissues. Pathway enrichment analysis predicted that circRNAs may regulate IDD disease through cell cycle regulation and ECM-receptor interaction. Guo et al. (152) sequenced IDD and normal tissues, revealing that 134 circRNAs were upregulated and 4 circRNAs were downregulated in IDD samples. Song et al. (153) reported that a total of 792 circRNAs were differentially expressed in a microarray hybridization analysis of the nucleus pulposus from patients with IDD, including 428 upregulated and 364 downregulated circRNAs. The authors selected circRNA_104670, which exhibited the most significant difference, to predict its downstream target as miR-17-3p. The AUCs of the two tests for the diagnosis of IDD were 0.96 and 0.91, respectively. Both values indicated high diagnostic efficacy, suggesting that they could serve as molecular markers for IDD diagnosis in the future.

Chen et al. (154) indicated that nucleus pulposus tissues from patients experiencing IDD exhibit high levels of circGPATCH2L expression. They found that circGPATCH2L is involved in DNA damage repair and subsequent apoptosis in NPCs. Mechanistically, circGPATCH2L acts as a protein decoy for tripartite motif containing 28 (TRIM28) within the aa 402–452 region. This action prevents the phosphorylation of TRIM28 and inhibits P53 degradation, leading to DNA damage accumulation, cellular apoptosis, and the progression of IDD. Xu et al. (155) discovered that disrupting the balance between autophagy and apoptosis in nucleus pulposus cells in the intervertebral disc also promotes IDD. Studies have shown that circPTK2 absorbs miR-193a-5p, miR-196b-5p, and miR-532-5p as sponges, resulting in reduced autophagy of nucleus pulposus cells and decreased self-protective ability. This process promotes apoptosis and activates circSMARCC1, circSLC30A7, circFRYL, and other circRNAs, ultimately contributing to the progression of IDD.

Since the intervertebral disc functions as a daily compressing and cushioning organ, some researchers have also suggested that the compressive load on the intervertebral disc could be the triggering factor for IDD (156). Excessive compression may lead to an imbalance between anabolic and catabolic processes of the ECM and apoptosis of the nucleus pulposus (157). Xiang et al. (158) simulated the pathological state of IDD *in vivo* by subjecting isolated nucleus pulposus cells to static compression of 1.0 MPa. Subsequently, a microarray was used to analyze the changes in circRNA expression levels before and after injury to the nucleus pulposus cells. It was found that a total of 1498 circRNAs exhibited decreased expression levels. The circRNA-CIDN, which exhibited the most significant decrease in expression, was validated in functional assays to promote ECM anabolism (proteoglycans, type II collagen), inhibit ECM catabolism (MMP-1, MMP-13), and reduce apoptosis of nucleus pulposus cells. These findings suggest that circ-CIDN may mitigate the progression of IDD by preserving ECM balance and suppressing nucleus pulposus cell apoptosis. These novel mechanisms and hypotheses offer new therapeutic strategies for preventing and treating IDD in the future.

IDD involves the loss of nucleus pulposus cell functionality and ECM integrity. CircRNAs, such as circRNA_104670, mediate ECM-receptor interactions, influencing pathways that regulate cellular senescence and ECM remodeling. These findings highlight circRNAs as pivotal regulators of molecular processes that underlie IDD progression, providing insights into potential therapeutic interventions.

### 5.6 CircRNAs and other orthopedic conditions

Emerging evidence suggests that circRNAs play significant roles in other orthopedic diseases, such as bone nonunion and heterotopic ossification (HO). Bone nonunion, characterized by the failure of a fracture to heal properly, has been linked to dysregulated circRNA expression. For instance, circRNAs modulate osteoblast and osteoclast activities, potentially influencing bone repair mechanisms and fracture healing. Similarly, HO, a pathological condition marked by ectopic bone formation in soft tissues, has been associated with aberrant circRNA-mediated regulation of inflammatory and osteogenic pathways.

In bone nonunion, the circRNA-miRNA-mRNA axis may interfere with key genes involved in osteogenic differentiation and bone regeneration. Previous research (115) highlighted the intricate role of circRNAs in modulating apoptosis and inflammation, which are critical in the progression of bone nonunion. In HO, circRNAs may promote aberrant osteogenesis by regulating inflammatory signaling and osteoprogenitor differentiation, as evidenced previously (159). These discoveries suggest that targeting specific circRNAs could serve as a potential therapeutic approach for controlling ectopic bone formation.

## 6 Clinical perspectives and challenges

Whether serving as a marker for clinical diagnosis, disease monitoring, and prognosis, or as a specific target for disease treatment, the clinical application of circRNAs shows promise. However, numerous difficulties and challenges still persist.

### 6.1 Molecular markers

First, circRNAs can serve as clinical biomarkers for diagnosing and predicting the prognosis of orthopedic diseases through tissue and cytology detection. Several studies have highlighted the potential of circRNAs as diagnostic markers, many of which can be identified through sampling peripheral blood or tissue from the primary disease site. To enhance the utility of circRNAs as diagnostic markers, efforts should focus on transitioning from invasive to noninvasive tests. Research should be directed toward advancing the detection of circRNAs in saliva, urine, plasma, and exosomes. Second, there is an urgent need to address the challenge of efficiently detecting and accurately quantifying circRNAs in a limited number of noninvasive samples to improve the practicality of circRNAs as diagnostic markers.

### 6.2 Therapeutic targets

Second, circRNAs also present opportunities and challenges as therapeutic targets for bone-related diseases. Several critical issues necessitate immediate attention.

① The regulatory mechanism remains unclear. The molecular adsorption sponge function of the currently popular miRs is just the beginning of the pathogenic mechanisms. In recent years, molecular mechanisms such as translation and protein synthesis by circRNAs, binding and regulation of RNA-binding protein (RBP) stability, and regulation of selective mRNA cleavage have been successively reported. Further research is essential to determine whether they are also involved in regulating common orthopedic diseases.

② Expression techniques. Most circRNAs are expressed at very low levels *in vivo*. Even for some circRNAs with relatively high expression abundance, achieving therapeutic concentrations is challenging. Overexpressing specific circRNAs *in vivo* remains a major challenge, and the most viable method is synthesizing exogenous circRNAs suitable for humans *in vitro* on a large scale. Liu et al. (160) connected five repeating linear fragments of circRNA scRNA21 containing miR-21 binding sites. They synthesized scRNA21 *in vitro*, which can bind miR-21, and confirmed in cell experiments that its biological functions were consistent with those of endogenous scRNA21. This approach to synthesizing individual downstream circRNAs is innovative, but it also has a critical flaw. This is because the regulation of circRNAs and miRNAs is not one-to-one but many-to-many, forming a complex competing endogenous RNA (ceRNA) network.

③ Delivery mode: Selecting the appropriate vector and delivering exogenous circRNAs to the precise site of disease occurrence remains unclear. Some studies have suggested using exosomes as a delivery method. Li et al. (59) demonstrated that packaging the oncogenic circ-0000190 into exosomes and transferring them from normal cells to OS cells inhibited their malignant biological behaviors of proliferation and invasion. The lyophilized exosome powder (161) is a well-established technology that can preserve exosomes for an extended period while maintaining their biological activity and stability. This preservation technique enhances the clinical application of exosomes as carriers of circRNAs. Exosomes can serve not only for systemic drug delivery but also for local drug delivery by transporting other carriers, such as hydrogels. Tao et al. (162) encapsulated exosomes containing circRNA3503 into a hydrogel for intra-articular injection to achieve localized drug delivery in OA. The gradual degradation of the hydrogel facilitated the sustained release of exosomes for a slow drug release. Apart from exosomes, other carriers like polymeric nanocarriers, mesoporous silica nanocarriers, and graphene nanocarriers also hold promise as carriers for circRNAs. Overcoming challenges such as achieving stable carriage of circRNAs, targeted and timed release, and carrier targeting are still areas that need to be addressed.

④ Decomposition and metabolism: circRNAs have a significantly longer half-life compared to linear RNAs because of their slow degradation process. The future implications of drug accumulation, degradation, and metabolic clearance in organs, as well as their impact on humans, remain unknown and have not been addressed in the literature. Subsequent research on the pharmacokinetics, pharmacodynamics, and toxicology of circRNAs in both animals and humans should be carried out accordingly.

⑤ Clinical utility. Current research combines clinical samples and basic experiments to identify circRNAs with potential therapeutic effects. Numerous studies have reported hundreds of circRNAs involved in orthopedic-related disease processes. However, the question of how to select the most therapeutic circRNAs as clinical targets remains unanswered. CircRNAs typically exert their biological effects through the circRNA/microRNA/downstream gene regulatory axis. Therefore, why are circRNAs chosen as therapeutic targets instead of downstream microRNAs or genes? These questions can only be answered through clinical studies to confirm the accuracy of the underlying experiments. This will necessitate a significant number of Phase I, II, and III clinical trials to systematically evaluate the feasibility of circRNAs as targeted drugs.

## 7 Summary and outlook

Given the stable configuration of circRNAs characterized by loop closure and considerable species conservation, coupled with the emergence of advanced high-throughput screening technologies for circRNAs, numerous contemporary studies have indicated that circRNAs are critical in the initiation and development of various orthopedic diseases. With ongoing elucidation of the functions and mechanisms of circRNAs in the realm of orthopedic diseases, circRNAs have great clinical significance as prognostic markers for the diagnosis of bone diseases and as new targets for individualized therapy. In conclusion, the mystery of circRNAs is gradually being solved, and circRNA-based diagnostic and therapeutic approaches will definitely play an important role in the clinical practice of bone diseases.
